# PATTERNS OF FAMILY CAREGIVING FOR OLDER ADULTS: THE ROLES OF PRIMARY AND SECONDARY CAREGIVERS

**DOI:** 10.1093/geroni/igad104.1217

**Published:** 2023-12-21

**Authors:** Rahul Malhotra, Jeremy Lim-Soh, Pildoo Sung, Ha-Linh Quach

**Affiliations:** Duke-NUS Medical School, Singapore, Singapore; Duke-NUS Medical School, Singapore, Singapore; Duke-NUS Medical School, Singapore, Singapore; Duke-NUS Medical School, Singapore, Singapore

## Abstract

Family members are often front-line carers for older adults. With research studies focusing largely on the primary family caregiver, less is known about how caregiving responsibilities are shared between family members. Using latent class regression, we described the prevalence of distinct patterns of family caregiving for older adults, in terms of who (division of assistance between the primary caregiver and secondary caregivers) provides what assistance (three types: with (a) activities of daily living; (b) health and social services; and (c) other socio-emotional needs), and their correlates. Primary family caregivers of 277 older adults (75 years and older) with functional limitations in Singapore, were interviewed in 2019-2020. Three patterns of family caregiving were identified. The ‘Shared-Diverse’ (43%) pattern typically involved multiple family caregivers providing all three types of assistance. The ‘Shared-Informal’ (27%) pattern also typically involved multiple family caregivers but no provision of assistance with health and social services; such care-recipients were more likely to be female (versus the Shared-Diverse pattern). The ‘Solo-Diverse’ (30%) pattern typically involved the primary family caregiver solely providing all three types of assistance; such caregivers were less likely to be working. Primary family caregivers in the Solo-Diverse pattern, versus the Shared-Diverse pattern, had higher depressive symptoms. The predominance of caregiving patterns involving multiple family members highlights the need for expanding family caregiving research, policies, and programs beyond primary family caregivers. The findings also underscore the importance of solo family caregivers, whose employment ability and mental health may be affected by their caregiving duties.

